# An Overview of Duplicated Gene Detection Methods: Why the Duplication Mechanism Has to Be Accounted for in Their Choice

**DOI:** 10.3390/genes11091046

**Published:** 2020-09-04

**Authors:** Tanguy Lallemand, Martin Leduc, Claudine Landès, Carène Rizzon, Emmanuelle Lerat

**Affiliations:** 1 IRHS, Agrocampus-Ouest, INRAE, Université d’Angers, SFR 4207 QuaSaV, 49071 Beaucouzé, France; tanguy.lallemand@inrae.fr (T.L.); martin.leduc@etud.univ-angers.fr (M.L.); claudine.landes@inrae.fr (C.L.); 2 Laboratoire de Mathématiques et Modélisation d’Evry (LaMME), Université d’Evry Val d’Essonne, Université Paris-Saclay, UMR CNRS 8071, ENSIIE, USC INRAE, 23 bvd de France, CEDEX, 91037 Evry Paris, France; carene.rizzon@univ-evry.fr; 3 Université de Lyon, Université Lyon 1, CNRS, Laboratoire de Biométrie et Biologie Evolutive UMR 5558, F-69622 Villeurbanne, France

**Keywords:** gene duplication, bioinformatic tools, paralogous genes, genome evolution, synteny

## Abstract

Gene duplication is an important evolutionary mechanism allowing to provide new genetic material and thus opportunities to acquire new gene functions for an organism, with major implications such as speciation events. Various processes are known to allow a gene to be duplicated and different models explain how duplicated genes can be maintained in genomes. Due to their particular importance, the identification of duplicated genes is essential when studying genome evolution but it can still be a challenge due to the various fates duplicated genes can encounter. In this review, we first describe the evolutionary processes allowing the formation of duplicated genes but also describe the various bioinformatic approaches that can be used to identify them in genome sequences. Indeed, these bioinformatic approaches differ according to the underlying duplication mechanism. Hence, understanding the specificity of the duplicated genes of interest is a great asset for tool selection and should be taken into account when exploring a biological question.

## 1. Introduction

The eukaryotic genome organization is complex and contains different types of sequences with much of them being non-coding sequences that may have an important impact on genome functioning and regulation. Moreover, genomes are highly dynamic with several ongoing processes allowing the creation of genetic novelty necessary for species to evolve and adapt to changing environments. Among the different possibilities, gene duplication is a very important mechanism providing new genetic material and opportunities to acquire new functions [1].

In particular, numerous examples have described the role of duplication in some cases of adaptation to environmental conditions [2]. For example, gene duplication has played a role in nutrient transport under stress conditions, in protection against heat, cold, or salty environments, in the resistance to drugs and pesticides, but also in the adaptation to domestication. Gene duplication can also be involved in speciation, especially via whole genome duplication (WGD) as it is suspected in plants, where a correlation has been observed between WGD and increased rates of speciation or divergence [3]. In particular, this mechanism is thought to have generated the new flowering plant *Mimulus peregrinus* within the last 140 years [4]. Although less numerous than in plants, some examples also exist in animals such as in *Drosophila* where the hybrid-male sterility gene Odysseus was formed by gene duplication [5]. On the other hand, duplication may also have important deleterious effects in humans and can be associated with some diseases [6]. For example, the analysis of human genes linked to diseases made it possible to show that 80% of them have been duplicated in their evolutionary history, the disease-associated mutation being associated with only one of the duplicated copies [7]. Recently, the analysis of the evolution of cancer suppression in mammals revealed that species known to be resistant to cancer contain the most cancer gene copies [8].

Duplicated genes are also called paralogs in contrast to orthologs, to refer to their homologous relationship, i.e., the fact that they descend from a common ancestor via a duplication event rather than a speciation event. The terminology concerning duplicated genes can be complex and depends upon different factors (for a review, see [9]). In particular, it may be difficult to assess precisely the evolutionary relationships between duplicated genes since duplication is often followed by speciation and gene loss. Several definitions have been proposed to integrate more or less precise ideas concerning the mechanism of formation and the evolutionary relationship among paralogs. For example, ohnologs correspond to paralogs that have been created by WGD [10]. Three new definitions, pro-ortholog, semi-ortholog, and trans-homolog, were proposed to account for situations in which one or both lineages that lead to two present-day genes involve gene duplication [11]. In that respect, a pro-ortholog is a gene that is orthologous to the ancestor of a set of paralogs of the gene under consideration whereas a semi-ortholog is one of the descendants of an ortholog of the gene under consideration, after that gene has duplicated. Trans-homologs can be defined as genes related to each other via two independent duplication events from the same ancestral gene. Moreover, it is also possible to link paralogous relationships to speciation events with the definition of in-paralogs and out-paralogs [12]. When paralogs from a given lineage have evolved by gene duplication that happened after a speciation event, they can be referred to as in-paralogs. On the opposite, paralogous genes which have evolved by duplication events happening before a speciation event, can be referred to as out-paralogs. Many other terms, although less used, have been proposed to take into account chromosomal position retention, the combination of vertical and horizontal transmissions or to highlight paralogous genes appearing to be orthologs due to differential gene loss [9].

In a genome, duplicated genes can thus be formed by various mechanisms and may have different ages and fates. This makes their bioinformatic identification all the more difficult since according to the methods used, different duplicated gene datasets will be identified inside the same organism. In this review, we thus aim at describing the evolutionary processes implicated in the formation and the fate of duplicated genes as well as the different bioinformatic approaches that can be used to identify them in genome sequences. The question of deciphering the evolutionary relationships among duplicated genes will not be discussed in detail, for reviews on the subject see [13,14].

## 2. Evolutionary Processes Leading to the Formation and the Fate of Duplicated Genes

### 2.1. How to Make New from Old: Duplication Mechanisms

**Figure 1:**
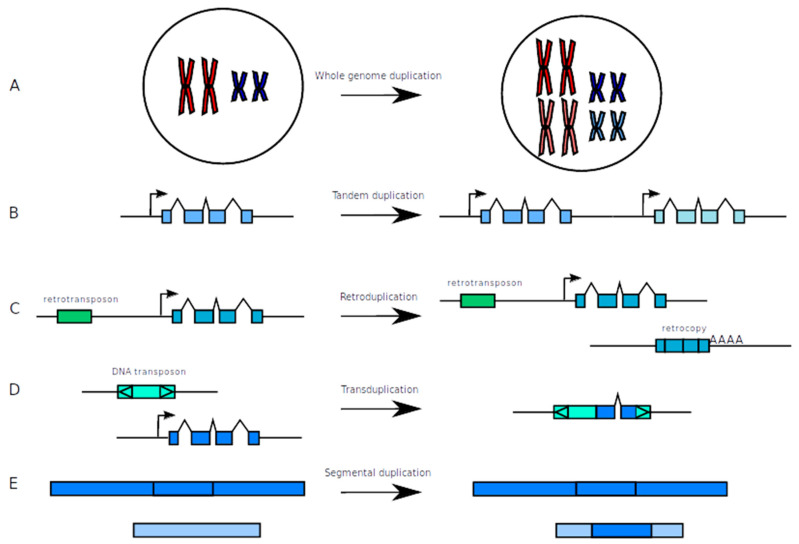
The different types of duplications. (**A**) Whole genome duplication which implies complete chromosome duplication. (**B**) Tandem duplications which produce identical adjacent sequences. (**C**) Retroduplication, which produces a retrocopy of a gene devoid of introns and with a polyA tail. (**D**) Transduplication in which a DNA transposon acquires fragments of genes. (**E**) Segmental duplications which correspond to long stretches of duplicated sequences with high identity.

Duplicated genes can appear under various forms which are the consequences of the mechanisms that generated them. Some of the mechanisms can be particularly well documented but it is not always the case, at least for some organisms. According to the mechanism, the results concerning the gene content can be different since it can either involve individual genes or all genes on entire chromosomes (Figure 1).

#### 2.1.1. Whole Genome Duplication (WGD)

In the first mechanism, duplicated genes arise from the duplication of complete chromosomes, which correspond to what is called whole genome duplication (WGD) (Figure 1A). In that case, all chromosomes from a genome will be duplicated, leading each gene from the genome to exist in two copies. This type of duplication has been well documented in plants and is defined as polyploidization, for which it is possible to distinguish the mechanism of hybridization between different species (allopolyploidization) or inside a given species (autopolyploidization) [15]. Different mechanisms have been shown to produce this outcome such as polyspermy, non-reduced gametes or incomplete mitosis during the early stage of embryo development [16]. Gene duplication, independently of the mechanism of formation, is largely present in plant genomes since on average 64.5% of genes have been recognized as duplicated in an analysis that considered 41 genomes and used the same methodology to build gene families, with a range going from 45.5% in a moss to 84.4% in the apple tree [17]. It is possible to estimate that several WGD events took place during the evolution of plant species, the most ancient happening in the ancestor of all seed plants about 319 million years ago and another more recent before the diversification of angiosperms 192 million years ago [18]. A large number of WGD are also consecutive to recent events. For example, the wheat group has evolved through different complex hybridizations among species from the plant genera Aegilops and Triticum followed by genome doubling. The most recent event giving birth to allotetraploid wheat (two different diploid parental species) has been proposed to occur about 300,000 to 500,000 years ago, while an allohexaploid wheat (three different diploid parental species) was formed only about 10,000 years ago [19]. Another domesticated plant, the Oilseed crop (*Brassica napus* L.) originated between around 6700 to 51,000 years ago by hybridization between two species, which were themselves polyploids [20,21,22,23]. The consequences of the different types of hybridization, and thus WGD, are that many plants arising from these processes have very large genomes. On the contrary, in other organisms, there are still some debates concerning the occurrence of WGD versus several more local duplications. This is the case in vertebrates in which the “2R hypothesis”, originally proposed by Susumu Ohno [1], assumes the existence of two rounds of WGD in their early evolution. The “2R hypothesis” has been the subject of numerous studies to prove this theory. This has led to numerous works published during the last twenty years either in favor of the “2R hypothesis”, or in favor of only one round of WGD, or rejecting any idea of WGD (see for a review [24]). The main reason explaining the difficulty to determine whether two rounds of WGD happened or not very anciently comes from two phenomena which could blur the signal. Both phenomena make it harder to detect ancient WGD either through the loss of signal (fractionation) or increased complexity (diploidization). The fractionation is characterized by a heavy loss of duplicated genes following WGD [25]. The diploidization refers to the chromosomal rearrangements and segment loss often observed after WGD when the genome goes back to a diploid state [26]. Indeed, a return to diploidization involves the transition to disomic inheritance as it has been proposed in Salmonid species, for example [27]. In a recent work, 61 animal genomes were used to reconstruct the gene order of the ancestral Amniota genome, to identify duplicated genes produced by the 2R in this genome, and to reconstruct the timeline of events conducting a pre-vertebrate genome going from 17 chromosomes to 54 after the occurrence of two successive WGD [28]. Although a lot of arguments seem now to be more in favor of the “2R hypothesis”, the question is still not completely resolved. Very recently, an investigation using phylogenetic approaches and tree topology comparisons of gene families containing at least three members and located on several human chromosomes led to the conclusion that small-scale duplication (SSD) events scattered on all the animal history were more likely to be involved in vertebrate genome evolution rather than WGD [29].

#### 2.1.2. Tandem Duplications

At smaller scales, local events called tandem duplication, create a novel copy of a gene next to it producing tandemly arrayed genes (TAGs) (Figure 1B). The molecular mechanism involved consists in unequal crossing overs, which can produce regions containing one or several genes, depending on the position of the breakage on the chromosomes [30]. These unequal crossing overs are either the result of homologous recombination between sequences (on homologous chromosomes or on sister chromatids) or of non-homologous recombination by replication-dependent chromosome breakages [31]. When multiple occurrences of unequal crossovers happen, it might lead to increasing or decreasing copy numbers in gene families. The molecular mechanism allowing the recombination depends on the sequences that promote the exchange between chromosomes or chromatids, which can be long direct repeats (>100 bp) and short ones (>12 bp) [32]. When repeats are long, the tandem duplication can arise via the homologous recombination whereas when they are short, duplication arises by single-strand annealing, template switching, or non-homologous end joining. This type of duplication leads to the formation of clusters of duplicated genes sometimes representing specific gene families. For example, this mechanism has been shown to confer soybean resistance against cyst nematode (*Heterodera glycines*) at *Rhg1*, a quantitative trait locus on chromosome 18, by changing the copy number variation that increases the gene expression [33]. In maize, thousands of tandem gene duplicates were identified that correspond to about 10% of the annotated genes [34]. Some of them may contribute to a phenotypic variation such as the *White Cap locus*, which provided the possibility to select white-grain color [35].

#### 2.1.3. Duplications Via the Action of Transposable Elements

Duplicated sequences can also be formed by the action of transposable elements (TEs) according to different ways. TEs are repeated sequences with the ability to move from one position to another along and across chromosomes and which may represent a very large proportion in genomes, going from about 3% in yeast to more than 80% in maize [36,37]. When they are mobilized, some of them can drag host sequences with them or can target the gene transcript, all of these having the consequence to duplicate the host sequences. There are two mechanisms by which TEs can promote duplication of complete genes or part of genes as a direct consequence of their transposition: The retroposition and the transduplication (Figure 1C,D). The retroposition mechanism consists of the reverse transcription of a messenger RNA from a host gene into a cDNA then inserted in another location of the genome by the action of the enzymes of a retrotransposon [38]. Genes submitted to this mechanism are located in the 3′ side of retrotransposons and benefit from a transcription read-through initiated inside the TE [39,40]. This new gene, that is called a retrocopy, has particular features such as the presence of a polyA tail in its 3′ end, the loss of introns, and the presence of target site duplication at both extremities which are the signature of its insertion. Retrocopies have been discovered in different organisms such as in mammals, and especially in the human genome where thousands of them have been identified [41,42]. Although less numerous, retrocopies have also been identified in insects such as in *Drosophila melanogaster* [43,44], or in the mosquito *Anopheles gambiae* [45]. Interestingly, it has been observed a bias in the location in the genome of these retrocopieswhich move from the X chromosome toward the autosomes in the insects [43,45]. In mammals, X chromosomes seem to have generated and recruited more retrocopies than the other chromosomes [46]. This type of duplicated gene is also found in plant genomes. For example, in the rice genome (*Oryza sativa*), between 491 and 1235 retrocopies were identified according to the methodology [47,48]. In *Arabidopsis thaliana,* 271 retrocopies were identified [48]. The other mechanism that involves TEs, the transduplication, happens when DNA transposons incorporate unspliced fragments of different genes, although the true mechanism is still unknown [49]. The gene fragments may still contain introns. First discovered in maize, this mechanism has then been documented only in plants such as *A. thaliana*, Japanese morning glory, soybean, and rice [49,50,51,52,53,54]. In rice especially, a particular type of DNA transposons called Pack-MULE, which represent about 3000 insertions in the genome, has been shown to contain sequence fragments derived from more than a thousand genes [54].

#### 2.1.4. Segmental Duplications

At a larger scale, segmental duplications, also called “low copy repeats”, correspond to very long stretches of duplicated sequences that can span between 1 to 200 kb and that share a sequence identity higher than 95% (Figure 1E; [55]). They have been first observed in several eukaryotic organisms such as the yeast [56] and humans [57]. These duplications are formed from the replicative transpositions of small portions of chromosomes. However, the exact mechanism is unclear and the fact that these duplications do not generate tandem repeats and that no short direct repeats at junction have been found suggests that neither unequal crossing-overs nor double-stranded breakages followed by repair are involved [55]. It has been proposed that in yeast, the segmental duplications could result from replication accidents [58] and that most of these sequences present a certain level of instability that can be rescued when translocation within another chromosome happens [59]. In *Drosophila*, high enrichment in TEs at segmental duplication extremities have been observed, indicating their possible implication in the duplication formation by homologous repair ends [60]. Similarly in mammals, particular types of TEs were found to be enriched at the junction of segmental duplications [61,62]. In the human genome, the sequence divergence of the duplicated segments has been used to estimate their evolutionary age which corresponds to the divergence between the New and Old World monkeys, 35 million years ago [63]. Segmental duplications account for an average of 13.7% of the total human genome, located in pericentromeric and subtelomeric regions [64]. Moreover, some chromosomes seem to be enriched in duplicated segments of this type such as the Y chromosome where they represent 50.4% of this chromosome [64]. In other mammals such as rat, mouse, or dog, this type of duplication is less abundant [64]. The comparative analysis of several genomes of Lepidoptera species made it possible to determine a large variation in the content of segmental duplications, going from 1.2% in the silkworm (*Bombyx mori*) to 15.2% in the postman butterfly (*Heliconius melpomene*) [65].

#### 2.1.5. Differences among Duplication Types

Notable differences depending on the formation mechanisms in terms of function, expression, evolutionary constraints, and protein interactions have been reported. For example, in yeast duplicated genes issued from WGD are associated with different sets of functions when compared to duplicated genes generated by SSDs [66,67]. This has also been shown in plants [68,69,70,71,72]. In *Arabidopsis* and rice, for example, TAGs were found to be enriched with genes that encode membrane proteins and with functions in “abiotic and biotic stress” when compared to other duplicated genes. TAGs were also underrepresented in genes involved in transcription and DNA or RNA binding functions compared to non-TAG duplicated genes [73]. More recently, Acharya et al. [74] reported a higher multifunctionality, estimated by the number of GO and Pfam annotations, for WGD duplicated genes compared to SSD genes in humans. They also observed a significantly higher proportion of essential genes among the WGD genes relative to SSD genes.

It has also been observed that duplicated genes differ in divergence of expression according to the mode of duplication. In *Arabidopsis* and in poplar, for example, WGD genes were found to display a lower divergence of expression than other duplicated genes [71,75]. In a study deciphering more deeply the different types of duplicated genes, Wang et al. [48] observed that in *Arabidopsis* and rice, WGD genes and TAGs displayed a lower divergence of expression than proximal, retrotransposed dispersed, and DNA based transposed duplicated genes.

In a recent study in Angiosperms, WGD duplicated genes were shown to be under stronger constraints to diverge at the sequence and expression level relative to SSDs [76]. It has also been observed that among WGD genes, those that are also involved in local duplications showed higher non synonymous substitution rates (Ka) and selection rates (Ka/Ks) than nonlocally duplicated WGD genes indicating that they evolve faster [77].

When considering protein-protein interactions (PPI) networks, it has been observed that the fraction of shared PPI between paralogous genes was higher when the genes shared the same function and showed a higher co-expression [78]. Among duplicated genes, WGD gene pairs displayed a higher fraction of shared PPIs than other duplicated gene pairs [78]. Arsovski et al. [79] examined the density of *Arabidopsis* DNaseI footprints, which are locations of protein binding sites, in the 1000 bp flanking upstream and downstream sequences of duplicated genes. They found that WGD duplicated genes had more footprints than TAGs. Moreover, WGD duplicated genes formed denser and more complex regulatory networks than TAGs when genome-wide regulatory networks were analyzed.

In summary, mechanisms that can lead to the formation of duplicated genes are various. The fates encountered by the new duplicated genes are also distinct and may depend on several factors.

### 2.2. Evolutionary Fates of Duplicated Genes

#### 2.2.1. Pseudogenization and Neo-Functionalization

After their formation, duplicated genes can encounter various fates (for a complete review on this matter, see [80]). The most likely is the pseudogenization or the complete loss of one copy since only one gene copy will continue to be under purifying selective constraints for its current function, leaving the other one free to accumulate deleterious mutations. These pseudogenes can be conserved in the genome. For example, *A. thaliana* and the rice contain thousands of pseudogenes in their genomes [81]. In humans, the olfactory receptor gene families have been shown to be composed of between 60–70% pseudogenes whereas in dogs pseudogenes represent less than 20% in those gene families, explaining the reduced sense of smell in humans [82,83]. Sometimes, however, the process of mutation accumulation can drive to a completely different outcome. Different models of population genetics have been proposed to highlight evolutionary mechanisms explaining the different fates of duplicated genes allowing them to be maintained in organisms (for specific reviews on this subject, see [84,85]). It has been proposed that three main steps are needed for duplicated genes to be maintained: Phase 1 consists of the origin of a genetic change through mutation, phase 2 corresponds to the fixation period when the mutation segregates in the population, and phase 3 corresponds to the preservation period where the duplication is conserved. Although infrequent, a mutation can provide a new allele giving rise to a new function for the gene copy. If this function is advantageous, it will be subjected to distinct selective constraints leading to its fixation in the population, in a process called neo-functionalization. There are two models to explain this mechanism. The Dyhkhuzen-Hartl model proposes that the mutations at the duplicated gene are fixed by drift and later, during a change in the environment, the new gene will become advantageous for the organism [86], whereas the “Adaptation model” proposes that an adaptive mutation is fixed at one of the duplicated locus because it is immediately advantageous [85]. Various examples of neo-functionalization have been described. The analysis of the copper transporter gene family, which contains the two genes *Ctr1* and *Ctr2*, suggested that the metazoan *Ctr2* arose several hundred million years ago via a duplication event of the *Ctr1* genomic locus. The resulting *Ctr2* then lost the ability to transport copper but gained the ability to regulate *Ctr1* cleavage [87]. In mammals, the family of retinoic acid receptors (RARs), which play a role in the embryonic development, contains three duplicated genes, *RARα*, *β*, and *γ*, with *RARβ* having kept the ancestral *RAR* role, while the two others have diverged both in ligand-binding capacity and in expression patterns suggesting that neo-functionalization occurred at both the expression and the functional levels for these genes [88]. A wide transcriptomic analysis in maize made it possible to determine that 13% of all gene pairs generated by WGD have been submitted to regulatory neo-functionalization in leaves [89]. The analysis of a gene family containing three members in the *D. melanogaster* genome made it possible to show that the family was created by two rounds of tandem gene duplication in the last five million years and that the two new duplicated copies have diverged in function from the parental copy [90].

#### 2.2.2. Sub-Functionalization and Functional Redundancy

Alternatively to the possibilities of pseudogenization and neo-functionalization, the duplicated genes can be submitted to sub-functionalization. In this process, accumulation of mutations drives the subdivision of the ancestral gene function among the duplicated genes. The complementarity can come from a change in the regulatory sequences, leading the two copies to have different expression patterns that will recapitulate the ancestral one when taken together, for example [91]. Several models have been proposed to explain this mechanism. In the first model called duplication–degeneration–complementation (DDC) the two gene copies will acquire complementary functions through independent mutations, which will lead to the need of the two copies to fulfill the original function by drift rather than by selective constraints [91]. Another possibility is described by the “gene sharing” model in which the acquisition of two expression domains could predate the duplication, with each copy losing one of the two afterward [92,93]. A close model corresponds to the “specialization” model [94] which proposes that an ancestral function is split among paralogs that will be expressed in different tissues or developmental stages. These two last models predict that the duplication will be followed by advantageous mutations in all duplicated genes with positive selection patterns detectable in their sequences. Moreover, it is supposed that the ancestral gene is able to fulfill the function of all duplicated genes but not so well. Numerous examples of sub-functionalization have been identified in eukaryotes. For example, in mammals, the Agouti-melanocortin system is represented by the Agouti protein (ASIP) and the Agouti-related protein (AgRP) whose expression patterns with distinct physiologic functions were acquired through sub-functionalization such that the current expression pattern and function of each protein correspond to a subset of the ancestral gene [95]. In tomato, two members of the gene family encoding phytochromes, which are light receptors playing a role in plant development, exhibit both common and non-redundant functions suggesting that they have sub-functionalized since their duplication [96]. Finally, it is also possible for the two copies of a gene to be both maintained in the genome by dosage subfunctionalization, each expressing the ancestral function, leading to a functional redundancy [97,98]. A model proposed to explain this possibility stipulates that expression reduction could help the retention of duplicates and the conservation of their ancestral function [99]. Several cases have been identified such as, for example, two members of the mammalian *HOX* gene complex, *Hoxa3* and *Hoxd3*, implicated in the embryonic development, that have been shown to display a similar function in mice [100]. In the yeast *S. cerevisiae*, duplicated genes were shown to maintain functional redundancy for several million years [101].

#### 2.2.3. The Fates of Duplicated Genes Depend on Different Factors

These different fates can be conditioned by the mechanism that led to the formation of the duplicated genes. Indeed, it was suggested that tandem duplication could more often produce duplicated genes having differential partitioning of regulatory sequences which implies that both genes would be necessary to recapitulate the ancestral expression pattern [102]. In *A*. *thaliana*, it was proposed that pseudogenes are more often derived from tandem duplications although this could be a bias due to the higher proportion of this type of mechanism compared to others in this organism [70]. The fate of retrocopies is often to become pseudogenes because of the lack of regulatory sequences [38]. However, it is sometimes possible for retrocopies to recruit other regulatory sequences allowing them to develop a new function. The structure of these retrogenes is usually chimeric with coding or regulatory features not present in the original genes [43,103,104,105,106]. Moreover, it has been observed in mammals that retrocopies located on the same chromosome than their parental gene have more chance to remain active indicating a role for the genomic context to maintain their expression [107]. In plants, a positive correlation has been observed between the size of gene family and the number of pseudogenes, with large families being more subjected to gene loss [81]. However, the gene function is also an important factor in the fate of duplicated genes. Indeed, in *A. thaliana*, pseudogenes tend to have functional counterparts in disease resistance, specialized metabolism cell wall modification, and protein degradation, whereas transcription factor and receptor-like kinase gene families are devoid of pseudogenes [70,81]. Other factors may also influence the fate of duplicated genes such as the number of protein interactions [76,108] as well as particular structural features [109]. According to the organisms, the outcome and formation mechanism of duplicated genes can also be different. In human and mouse, for example, the relative contributions of two types of duplication mechanisms made it possible to show that tandem duplications contributed more to duplications in the entire genome than retroposition, except for the two-copy gene families, and generated duplicated genes with more chance to be retained [110]. At another scale in primates, recent duplicated genes originated more often from segmental duplication than in other mammals in which the main mechanism to generate them rather corresponds to tandem duplication [111]. WGD in humans was proposed to have generated duplicated genes functionally more divergent but with a higher proportion of essential genes, which is the opposite trend to what was observed in yeast [74]. In *Drosophila*, young duplicated genes were shown to be preferentially subjected to neo-functionalization, implying the retention of almost two-thirds of these duplicated genes [112]. In plants, where most duplicated genes are derived from WGD and tandem duplication, a functional bias can be observed in genes according to their mechanism of formation [70]. Thus, genes involved in responses to environmental stimuli and upregulated in stress conditions are rather generated by tandem duplication, which implies that this mechanism is important for adaptive evolution in changing environments. Recently, a model was proposed to explain the gene retention after WGD in *Paramecium* species by dosage constraints, i.e., the majority of duplicated genes keep their ancestral function and are retained to produce the requested amount of proteins to perform this ancestral function [98].

In the next section, we will present in detail some of the current bioinformatic methodologies available to identify and analyze duplication in genomes with the goal to emphasize their advantages and weaknesses according to the situation.

## 3. Bioinformatic Approaches to Identify Duplications in Genomes

The identification of duplication within or between genomes is a complex process. Many algorithms have been developed for this purpose and different approaches can be used that have different aims and computation costs. Moreover, some of them are more suitable to search for a particular duplication event, are more optimized for large genomes, can deal with multiple genomes, or can handle genomes that have undergone multiple duplication and rearrangement events. In addition, there may be difficulties in the installation, the configuration, the launch, and the parsing of the results. This means for the user that programming skills may be required to use some of these softwares. There are also variations in the input data and the pre-processing requirements, the computing time or the associated visualizations, all of this making the choice of a tool not easy. Moreover, these tools do not all identify the same type of duplication and may therefore, be more or less adapted according to the biological question investigated. In summary, there is no stand-alone software that can solve all these problems and the choice of the tool will depend on computer skills but also on the genomes being compared and the biological questions being asked. In the following sections, we will present the different types of algorithms highlighting their specificities, advantages and weaknesses, with a focus on some tools that will be presented in more detail.

### 3.1. Paralog Detection

As said before, homologs are genes that share a common ancestry and are divided between orthologs (derived by speciation) and paralogs (derived by duplication). Based on this definition, the search for duplicated genes can be done through the identification of paralogous relationships. Therefore, it can be conducted by either identifying homologous genes in a given genome, which by definition can only be paralogous, or between multiple genomes before distinguishing orthologs from paralogs. Several approaches exist to this aim that we will present below.

#### 3.1.1. Homology Assessment

Homology, even if defined by a few words, is a challenging concept to be detected through bioinformatic tools (for a broad overview, see [13,113]). The only material given to us to infer common ancestry that may have started millions of years ago is the sequences of contemporary organisms. A notable exception to this limit came with the rise of paleogenomics which aims at sequencing genomes of extinct species through preserved elements such as ancient seeds or fossilized body parts [114]. However, even if paleogenomics provides useful information, the amount of material is scarce compared to the number of contemporary species. Two methods are typically used to assess the homology between genes: The sequence similarity and the gene structure. Both methods rely on the idea that common ancestry (i.e., homology) is the most likely explanation when two genes share a strong similarity and/or structure. The limitations of these methods account for the aforementioned problem of inferring history through present traces: Divergence becomes difficult to detect when the distance between species increases. Hence, when two genes share sequence similarity or structure, it is a strong indication of homology, but when two genes do not share those, it hardly says anything about their homology.

The sequence similarity can be tested with a sequence alignment algorithm. The most popular ones such as *BLAST* [115], *Psi-BLAST* [116], and *HMMER3* [117] are heuristic methods. Thus, they might not give the best results, but they drastically save computational time compared to a classical method such as the Smith and Waterman algorithm [118] even though some implementations have tried to make it faster as *PARALIGN* [119] or *SWIMM* [120]. In the case of homology, the alignment is generally performed on protein sequence instead of the gene. This allows a greater sensitivity since amino acid substitutions occur less frequently than nucleotide substitutions allowing silent mutations and because introns generate a lot of noise [121]. With these methods, the homology is tested against a cutoff on three different metrics: E-value, bit-score, or percent identity. The e-value is a statistic representing the expected number of times a given alignment score would occur by chance given the length and number of sequences being aligned. It is the most widely used metric as a first step to assess homology. Since the e-value is dependent on the database size, a potential caveat when setting a cutoff is to apprehend how the results might change for different databases. The bit-score is another metric measuring the sequence similarity given the raw score and the score system used but independent of the length or the number of sequences being aligned. The bit-score might be preferred in the case of a comparison between alignments since it relies only on the two sequences being aligned. Finally, percent identity is a straightforward metric giving how many amino acids are identical in the local alignment. When assessing homology on a genome-wide scale, the difficulty resides in setting the right cutoff for these metrics. For instance, to capture duplicates that diverged in function, the threshold needs to be relaxed, but with the risk of increasing the number of false positives. Based on empirical results, a 30% identity is generally accepted as a significant cutoff for protein homology [122]. However, countless identified homologs have an identity percentage lower than 30%. The same problem arises when using only e-value or bit-score. To allow better identification, more complex similarity-based metrics were developed. For example, Rost [123] proposed a formula based on the homology-derived secondary structure of proteins (HSSP) curve defined by Sander and Schneider [122] and considering the number *L* of aligned residues between two proteins to define a curve to separate true and false positives. Two proteins are then considered homologous if the proportion *p* of identical residues over *L* aligned residues is higher than the cutoff point defined by the formula. Li et al. [124] proposed a rewording of Rost’s formula to define different sets of duplicated genes with different stringencies in human. Since a gold standard cutoff is impossible to determine, a variety of values are used, sometimes combining different metrics leading to different results (Table 1).

When not working on a genome-scale but on specific sequences, homology imputation can be reinforced by looking at the gene structure. Domains shared by proteins are strong indicators of homology. Conserved domains can be found in databases such as Pfam [132] or InterPro [133] and searched against sequences of interest. This method is also a great tool to unravel complex evolution such as gene splitting and fusion for multi-domain proteins but require a time consuming manual expertise.

When searching for duplicated genes within a genome, assessing homology inside this genome is enough. However, when comparing multiple genomes, a link needs to be made between homologs of the different genomes. This raises the issue of resolving ortholog and paralog relationships. For this, a different kind of method needs to be applied. At first, methods to identify orthologous genes were only constructing orthologous groups because they focused on one-to-one ortholog relationships across multiple species. However, with the addition of one-to-many and many-to-many relationships, paralogs were included. Therefore, it could be argued that these methods are eligible to detect duplicated genes across multiple genomes. They are generally split into two categories: The graph-based methods and the tree-based methods [14,134]. Generally, graph-based methods construct a homology graph then build clusters of genes based on the types of inferred relationships. On the contrary, tree-based methods identify clusters of genes before constructing a tree along which the types of relationships are inferred.

#### 3.1.2. Multispecies Graph-Based Methods

In graph-based methods, each gene is a vertex and a homology relationship is depicted by an edge. These edges are first drawn by assessing sequence similarity in the various forms described before. At this step, edges only correspond to potential homology relationships which can be orthology, paralogy, or noise. The noise can be removed by the clustering step. Depending on the clustering method, some paralogous relationships can also be removed. It is important to note that for the resolution of ortholog and paralog relationships, all these methods consider that for a given speciation event, in-paralogs are less diverged than orthologs that are less diverged than out-paralogs.

One of the first proposed clustering methods was the identification of triangle patterns inside a graph where at least three genomes are used [135]. It relies on the idea that two similar genes from two genomes, which are also similar to a third gene from another genome are highly susceptible to be orthologs. Then, triangles sharing similar edges are added to the same group until no other can be added. These groups, called clusters of orthologous groups (COGs) can therefore contain paralogs. However, the nature of the paralogs included in a group is hard to control. Hence, another way to detect paralogs based on graph exploration was proposed with *InParanoid* [136]. Here, two genes from different species with a best reciprocal hit are defined as orthologs and will be used as a seed for the group. Any gene having a better score with the seed gene of the same species than with the seed gene from the other species is included inside the group as an in-paralog relative to the speciation event. Thus, only in-paralogs in regard to the speciation event considered should be added, allowing a better control over the group formation. The method *Hieranoid* expanded this idea with the use of a guiding species tree for a better scalability when using many species [137]. The algorithm enlarges groups by exploring the guiding tree. It first runs *InParanoid* between two closely related species. Then, it creates a pseudo-species where each identified homologous group is represented by either a consensus sequence or a Hidden Markovian Model profile, depending on the number of sequences. *InParanoid* is then used again between the pseudo-species and the next closest neighbor. The process is repeated until all species are included in the analysis. By keeping track of groups formed at each step, it is possible to identify the speciation event encompassing any in-paralog pairs. Acting as a synthesis between *InParanoid* and COGs, both *eggNOG* [138] and OrthoDB [139] start by identifying groups of in-paralogs for each species then link them between species using triangulation.

Considering that the *InParanoid* method was reliable to detect “ancient” paralogs but not “recent” ones, Li et al. added steps and proposed another method, *OrthoMCL* [140]. It begins by the same ortholog seed approach but with the constraint that in-paralogs must have a better score with the seed genes from their respective species than with any other sequences from any species. In addition, a Markovian Cluster algorithm is run to simulate a random walk on the graph with each edge having a transition probability depending on the similarity score. This makes it possible to identify robust subgraphs and notably separate diverged paralogs. Using also a similar approach than *InParanoid*, the method *OrthoInspector* starts by constructing species-wise in-paralogous groups. Inside a species, an in-paralogous group is inferred for each protein [141]. Inside a species, a group of potential in-paralogs is inferred for each protein. When two proteins are potential in-paralogs, only the intersection of their respective potential groups is conserved as the final in-paralogous group. Therefore, if we have three proteins A, B, and C, they will belong to the definitive in-paralogous group (A, B, C) if and only if all three potential in-paralogous groups constructed for each protein give (A, B, C). This stringent method creates groups of lowly diverged in-paralogs. In-paralogous groups or single proteins are then grouped between species based on best-reciprocal hits.

Finally, two other methods add an interesting consideration regarding homologs. Aiming to tackle a well-known problem of sequence alignment, *OrthoFinder* [142] allows a reliable incorporation of short sequences. Indeed, alignment score is correlated with the sequence length, which is a problem for short sequences giving high scores even when not related. *OrthoFinder* proposes a normalization of the alignment score after a grouping according to the sequence lengths into equally sized bins. This normalization makes the score for short and really long sequences less dependent on the sequence size. Another interesting method, *OMA* [143], proposes to detect falsely imputed orthology inferences due to paralogs with differential gene loss. The detection is performed by using a third species containing both paralogs which acts as an evidence of non-orthology. *OMA* is also more permissive in the grouping of paralogs because it takes into account that paralogs may evolve faster than orthologs [144].

When studying genes, especially across species, representing their evolutionary relationships as a tree is easier to analyze. However, constructing such a tree is done at the cost of computational time. In addition, different strategies can be adopted for the tree reconstruction.

#### 3.1.3. Multispecies Tree-Based Methods

In tree-based methods, homology is assessed according to the various forms described before, then groups of homologs are constructed across species. Genes from these groups are aligned to build gene trees. Paralog and ortholog relationships are then resolved by the reconciliation of the gene trees and the associated species tree. Therefore, in these methods, the detection of duplicated genes is only performed at the first step. The tree construction only influences the evolutionary history used to explain the appearance of such duplications.

In regards to the homolog grouping strategy, tree-based methods are generally more inclusive than graph-based methods. Indeed, after the group construction, they use all the sequences from all the species to infer paralog and ortholog relationships. Therefore, they can extract more information and are less restricted by false homology prediction and thus are able to capture more diverged homologs. Most of them construct homologous groups by clustering all genes that have a significant alignment score, defined differently according to the method used such as TreeFam [145], BranchClust [146], HOGENOM [147], or PhylomeDB [148]. Some tree-based methods use pre-processed homologous groups and are only used to reconcile the gene and species trees such as *Orthostrapper* [149], *Softparsmap* [150], or *LOFT* [151]. Therefore, graph-based methods can be used as an entry-point to combine the power of both methods.

When reconciling the gene and species trees, all these methods use the Maximum Parsimony principle [152]. This is translated by minimizing the number of duplication events, which are assumed to be rare events. A notable exception is *PrIME-GSR* [153] that tries to take into account the duplication and loss of genes through a probabilistic model. Apart from this exception, tree-based methods differ according to the type of species tree they accept, how they root the gene trees, and how tree uncertainty is assessed. Since it does not affect duplication detection, they are not as thoroughly explored as the graph-based method (for a complete review, see [14]).

### 3.2. Detection of Syntenic Blocks (WGD-Segmental Duplications)

A syntenic block can be defined as a region of the genome spanning a number of genes that are orthologous and co-arranged compared to another genome [154]. Two regions of a genome with a number of homologous genes co-arranged with each other can also be defined as a syntenic block. Here, we focus on this second definition because pairs of homologous genes between these pairs of regions correspond to duplicated genes.

It can be interesting to access different databases storing pre-calculated syntenic blocks shared between different species. This makes it possible for an easy and direct access to reliable information without any computation. Nevertheless, these databases cannot include every contemporary species nor information about recently released genomes. This implies that depending on the organism being studied it can be necessary to manually identify syntenic blocks using different tools. To accurately detect homologous chromosomal segments within a genome or between different ones, many approaches and tools are available. The choice of the tool depends on various parameters.

A first important parameter is the degree of preservation of duplicates in the compared genomes. This will influence the level at which the study should be conducted, and thus will impact the choice of the tool since each of them works at a particular level. For closely related genomes, synteny can be studied at the DNA level using tools such as *Satsuma* [155] or *SyMap 3.4* [156]. In the case of more distant genomes, the DNA level cannot be used because the sequences will be too divergent. A solution is to perform analysis at the protein level because coding genes may retain for a longer time enough amino-acid sequence similarity and a similar relative order along chromosomes. Tools such as *MCScanX* [157], *i-ADHoRe* [158], *CYNTENATOR* [159], or *SynChro* [160] search for syntenic blocks using protein sequences and can therefore be adapted to this type of genome comparison. Finally, in the case of more distant genomes, it is more appealing to use tools based on analyses at the protein level and on the construction of profiles, graphs, or statistical models to help manage the evolutionary distance.

Four types of approaches can be applied to search for syntenic blocks. The first one is based on the construction of a sparse matrix of homologous genes. The matrix is investigated to look for dense diagonals which correspond to the syntenic blocks. Tools such as *i-ADHoRe 3.0* [158], *DiagHunter* [161], *FISH* [162], *SyMAP* [163], or *Cinteny* [164] implement this type of approach. The second approach corresponds to different greedy algorithms that will be optimized by dynamic programming at the benefit of computational costs. This type of algorithm operates by constructing chains of collinear gene pairs, called anchoring genes. It is implemented in tools such as *DAGchainer* [165], *MCScanX* [157], or *LineUp* [166]. An important sub-category of this approach consists of algorithms based on aligning sequences using a modified Smith-Waterman algorithm as in *ColinearScan* [167] or *CYNTENATOR* [159]. To continue, the graph approach aims at building graphs allowing the identification of the syntenic blocks. To do this, local collinear alignments are constructed between the input genomes. By combining the local alignments with the blocks, a graph can be constructed which allows, after different analyses, the reconstruction of the syntenic blocks. This approach can be found in tools such as *DRIMM-Synteny* [168] or *Enredo* [169]. Finally, another approach aims at inferring syntenic blocks based on genomic rearrangements. This type of approach can be useful in the reconstruction of ancestral genomes and has been implemented in different tools such as *GASTS* [170] or *PMAG++* [171,172]. This approach is not covered in the present review but has already been discussed in another recent review article [173].

All these tools are able to answer different questions and their use depends on the number of studied genomes as well as the level of divergence among them. Most of them have many critical parameters, sometimes with important pre-processing requirements, which need to be mastered before obtaining reliable results. Most of the tools are presented in a comprehensive format in Table 2. Therefore, the purpose of this section is to examine in detail a representative sample of tools illustrative of each approach.

#### 3.2.1. Approaches Based on the Construction of Homologous Gene Matrices

These approaches correspond to tools based on the search for syntenies using clustering of neighboring matching gene pairs. The basic concept is to consider the homology in or between genomes as a sparse matrix. In summary, homologous gene pairs are considered as 1, other cases are encoded as 0. The goal is to detect syntenic regions by searching for dense diagonals of 1 in the matrix. Tandem duplication can also be accounted for by detecting horizontal or vertical lines of 1.

The main advantage of this approach is that it is designed around a formal definition of the syntenic blocks. Moreover, statistical validation can be performed on putative syntenic blocks to filter out false positives. However, several weaknesses exist for this approach. To begin with, the important impact of the parameters requires a good knowledge of the biological question asked. With this type of approach different parameters are critical and need to be finely tuned. An example being the size of the gap allowed between genes considered as belonging to the same block. This parameter can deeply affect the results, and needs to be configured according to the specificity of the study. The size of the gap depends mainly on the density of the matrix, i.e., the density of the pairs of homologous genes between the segments constituting the matrix. On one hand, a small gap value results in many small syntenic blocks that are more difficult to analyze. On the other hand, a high gap value produces long blocks that are easier to analyze but allow for more false positives. Moreover, the metrics used to estimate the distance between genes in a matrix are also an important setting. Two types of metrics are often proposed: The Diagonal Pseudo Distance (used by *i-ADHoRe* and *DiagHunter*), and the Manhattan Distance (used by *FISH*, *SynMAP,* or *Cinteny*). The Diagonal Pseudo Distance promotes genes near the diagonal axis and therefore, the distance inflates rapidly the further away genes are from this diagonal. In contrast, the Manhattan Distance tends to give smaller distances between aligned genes on the vertical or horizontal axis. Other types of distances have been implemented in tools such as *PhylDiag* [174] that uses the Euclidean Distance or the Chebyshev Distance in addition to the others mentioned above. Thus, the choice of the distance is not easy and will impact the results as surely as a wrongly set gap value. A benchmark analysis suggested that the Manhattan Distance gives the best results among these four distances [174]. The importance of the configuration is really to be taken into account when using this approach in an optimal way and makes these algorithms difficult to use without a minimum of expertise on both the tool and the biological question. Moreover, statistical tests to evaluate homologous regions are based on the assumption that the rate of gene loss is balanced between homologous regions. This is not the case for many species. Furthermore, some differences in terms of genome structure, especially the gene density and repetition in chromosomal regions, both locally and at the genome level, are difficult to account for with this approach. Finally, matrices can only compare genomes by pair, which implies that benefits of comparing multiple genomes at once, including WGD studies or diverged synteny blocks, cannot be done. Moreover, this approach cannot resolve multiple relationships between genes, detect inversions, nor non-overlapping syntenic blocks. To finish, not all of these implementations can detect tandem duplication. In the already cited tools, only i-*ADHoRe* and *FISH* can handle them. We will present these tools in more detail below.

##### i-ADHoRe (Iterative Automatic Detection of Homologous Regions) 3.0

*i-ADHoRe* [158] is one of the most used programs to find syntenic blocks and can be considered as a state-of-the-art algorithm. In its latest version, i-*ADHoRe* enables the detection of genomic homology through the identification of gene collinearity. This version is well optimized to handle a large number of genomes, taking advantage of parallel computing.

The algorithm begins by assimilating tandem duplicated genes as a single representative. Then, for each pair of genes, a sparse gene homology matrix is constructed. In this matrix, homologous genes are considered as dots, making collinearity zones seen as dense diagonals. Gene clusters with at least three homologous gene pairs are included in diagonals after a statistical validation taking into account the overall background density of the matrix. In the case of multiple clusters found, a correction for multiple testing is performed using the Bonferroni or False Discovery Rate (FDR) method. This part corresponds to the traditional homology matrix approach. The next part of the algorithm is an optimization by dynamic programming.

Significant collinear regions found during this initial detection are aligned using the progressive Needleman–Wunsch (pNW) algorithm or a greedy graph-based algorithm [178]. The results of this alignment are stored into a profile, which contains the combined content of the two collinear regions and constitutes a more sensitive probe to find new homologous regions including more degenerated ones. Using this newly constructed profile, a search is performed in an iterative way. Thus, this profile is used to search for new sequences that can be aligned with it. If possible, the new matches are added to the profile. As long as new collinear regions can be added to a profile, these two steps are repeated.

The results are provided as text files and two associated plots: A dot plot and a set of graphs representing each final aligned profile.

This tool has many key parameters that directly influence the quality of the results:

- prob_cutoff, is used to store the maximum probability for a cluster to be generated by chance. The default value is 0.001.
- gap_size, indicating the maximum distance between genes in a cluster. The default value is 15.
- cluster_gap, indicating the maximum distance between individual base clusters in a cluster. The default value is 20.
- q_value, storing the minimum *r*^2^-value which measures the quality of the linear regression prediction.
- anchor_points, the minimum number of anchor points which is comprised between 3 and 6.

The main advantage of this tool is to allow the computation of multiple genomes thanks to different optimizations including the use of parallel computation, an efficient statistical model to estimate *p*-values of diagonals before including them, the use of greedy graph-based alignment algorithms, and the use of ordered gene lists instead of genome sequence. This level of abstraction allows a more efficient detection of collinearity and thus the divergent intergenic sequences will have less impact on the algorithm.

##### OrthoCluster

In this category, *OrthoCluster* [177] appears as particular. It is not based on the classical approach of homology matrix construction although it is using the same philosophy by identifying syntenies via the clustering of neighboring matching gene pairs. This program is based on an algorithm implementing a strategy of tree enumeration to detect orthologous gene clusters. This tool can handle many genomes and makes it possible to overcome some of the weaknesses of the other classical approaches. Indeed, it can detect four types of genome rearrangements including insertions, transpositions, insertions/deletions, and reciprocal translocations via different algorithms. To detect reciprocal translocations (exchange of DNA parts by recombination), *OrthoCluster* merges syntenic blocks to build the longest possible blocks, identifying blocks not broken by duplications, inversions or transpositions. To detect transpositions (regions moved from a chromosome and inserted into a non-homologous chromosome), *OrthoCluster* searches in each adjacent syntenic block for a region between their homologous syntenic block in the other genome. If a fragment of less than 50 genes is found between them, a transposition is identified. Then, the detection of inverted segments in the genome is performed by checking if the order of the genes is the same in each syntenic block. If the gene order is inverted between the two, this region constitutes an inversion. Finally, in order to detect insertion or deletion of genes, *OrthoCluster* compares the pairs of adjacent syntenic blocks in the reference genome. Genes identified between these blocks are considered as insertions/deletions and reported. It can also detect segmental duplications and resolve one-to-many relationships. Moreover, the orientation and the order of genes are taken in to account. Nevertheless, this tool is limited to the orthology detection and can therefore only be applied on closely related organisms.

The fine-tuned configuration of this tool is crucial to obtain reliable results. Eight parameters can be defined by the user to set up the algorithm according to the needs:

- *l* max defining the upper bound on the number of genes in each cluster.
- *l* min defining the lower bound on the number of genes in each cluster.
- *op* maximal percentage of out-map genes allowed.
- *ip* defining the maximal percentage of mismatched in-map genes allowed.
- *op* and *ip* can control the number of genes involved in transpositions in synteny block.
- *i* maximal number of mismatched in-map genes allowed.
- *o* maximal number of out-map genes allowed.
- *r* to find order-preserving clusters.
- *s* to find strandedness-preserving clusters.

#### 3.2.2. Algorithms Using Dynamic Programming Optimizations

This type of approach generally implements algorithms more costly in computation than the homology matrix approaches. Some methods benefit from dynamic programming to build a chain of collinear pairwised genes. In these methods, the dynamic programming algorithms are implemented in the search for collinear genes, allowing an exhaustive and fast search. A scoring system is set up allowing to build pairs of adjacent collinear genes, which constitute anchoring genes, and to penalize the distance between them. The main advantage of a multi-alignment of collinear chromosomal regions is its ability to reveal past WGD events and complex chromosomal rearrangement relationships. In this type of approach, the syntenic blocks are composed of anchoring genes that are located at collinear positions and between them non-anchoring genes that are assumed to have undergone mutations. Nevertheless, the user needs to already know what to look for and the characteristics of the genomes and syntenic blocks being studied.

##### MCScanX and MultiSyn

*MCScanX* [154] is one of the most used tools aiming at searching for syntenic blocks and is implemented in the webtool *MultiSyn* [176], allowing biologists with no informatic skills to use this approach. Moreover, this tool produces additional visualizations allowing a simplified analysis.

The *MCScanX* algorithm takes place in three steps. The first step uses the results of an all-against-all comparison using BLASTP [179] to find collinear blocks. BLASTP matches are sorted according to their genomic positions. To handle tandem regions, all consecutive genes with a BLASTP match that are separated by less than five genes, are collapsed into a single representative. Then, the highest scoring chains of collinear gene pairs are searched for using dynamic programming. Non-overlapping chains involving at least five collinear gene pairs are saved. In a pair of collinear blocks, two distinct genomic locations with aligned collinear genes are assigned as anchors.

The second step makes it possible to assign each syntenic block to a gene class. To do that, all genes are first assigned to the singleton class. Genes with BLASTP hits to other genes are assigned to the class “dispersed duplicates”. If the hits are close enough, they are assigned to the class “proximal duplicates”. If the hits are neighboring, they are assigned to the class “tandem duplicates”. To finish, anchored genes are assigned to the WGD/segmental class.

In the last step, twelve downstream analyses can be performed using different scripts and correspond to the computation of the nonsynonymous and synonymous rates (Ka and Ks), the generation of various plots, the construction of gene families with associated analyses, the detection of collinear tandem arrays, the computation of the number of intra- and inter-species collinear blocks at each locus of reference genomes, and the display of statistics on gene numbers at different duplication depths.

To be functional this tool needs to be configured using at least six parameters:

- match_score, defines a threshold used to validate a synteny block. Default value is 50.
- gap_penalty, defines the penalty added when opening a gap. Default value is 21.
- match_size, defines the number of genes required to consider it as a collinear block. Default value is 5.
- e_value, defines the statistical significance of the synteny block alignment. Default value is 1e-10.
- max_gaps, maximum number of gaps allowed. Default value is 25.
- overlap_window stores the maximum number of genes to collapse BLAST matches. Default value is 5.

The special feature and strength of *MCScanX* is that each chromosome is used as a reference. Thus, all collinear segments in pairs are mapped. This is followed by a multiple alignment procedure of homologous genes, described as “transitive homology” [180]. This approach allows *MCScanX* to match regions that were not initially detected based on their collinearity with the reference.

To conclude, this tool is powerful and allows performing many analyses, if the user has the ability to install and configure it properly. *MultiSyn* eases the configuration step, the initial formatting of the data and the analyses using a graphical interface. As a final advantage, this tool can be deployed locally. As for *i-ADHoRe*, the use of ordered gene lists instead of a genome sequence allows getting more reliable results at lower computational costs.

##### SynChro

SynChro [160] is based on Reciprocal Best-Hits (RBH) to construct syntenic blocks. This algorithm is faster and easy to use thanks to its unique parameter (Δ) which represents syntenic block stringency. To go into more details, this algorithm is composed of three simple steps. In the first step, RBH are identified using *Opscan* (http://wwwabi.snv.jussieu.fr/public/opscan/), a tool based on the FASTA algorithm [181]. RBH can be defined as two genes whose best hit is mutual. In the second step, the RBH makes it possible to define syntenic blocks using co-localizing RBH (defined by Δ) along the chromosomes of two genomes as anchors. In the third step, syntenic blocks are completed by non-RBH homologs. Genes are defined as non-RBH if they share 30% of similarity and if the ratio of the length of the match between the two sequences and the length of the smallest sequence is greater than 0.5.

This tool provides various graphical outputs including dotplots, chromosome painting, and synteny maps, as well as text results. Therefore, it makes it possible to obtain in a limited computational time very good quality results with a fast handling in an “all in one” manner allowing to easily visualize the results.

##### CYNTENATOR

*CYNTENATOR* [159] is a tool aiming at identifying conserved syntenic regions between distant genomes. This tool is based on a progressive multiple gene order alignment. The main advantage of this tool is its scalability allowing it to work on more than 10 genomes contrary to many other approaches. Moreover, it makes it possible to get rid of heavy preprocessing steps due to its high flexibility.

To begin, a progressive pairwise alignment between genomes is performed. These alignments are based on a user-defined phylogenetic tree that directs the order in which the genomes will be compared. Only valid alignments gathering homologous regions of all species are retained for collinearity search in the next genome. This filtering step helps lower the computational costs and allows determining the gene order conservation between distant genomes. This step is followed by a pairwise alignment using a Smith-Waterman local alignment weighted by the phylogenetic distance. The results of these alignments constitute the syntenic blocks. The use of a progressive alignment algorithm makes it possible to conduct studies on several large genomes while taking into account the phylogeny of the studied species. The absence of a heavy pre-processing on the input data, except an all against all homology score, allows to avoid bias.

##### SyMap

*SyMap* [163] is a tool based on *DAGChainer* [165]. The advantage of this software is that its interface via a web application allows the user to be free from any configuration and data preparation via the code. In addition, this tool allows retrieving various additional information and in particular the Ka/Ks ratio using *PAML* [182]. The intermediate results can be retrieved and the final results can be visualized in an interactive dot-plot. Once the genomes have been added to the database and the parameters have been defined, the computations are launched. The *SyMap* algorithm works as follows. First, the genomes are aligned using an alignment software. Different tools can be used for this step, including BLAST. Then, different filters are applied and in particular the condensation of tandemly duplicated genes into a single occurrence and filtering out of repeated sequences. The syntenic blocks within this homologous matrix are then searched for using *DAGChainer*. Finally, different visualizations are constructed. The main advantages of this tool are its speed, the ease of use, and the visualizations. However, some parameters are not configurable and it does not allow the study of more than two genomes at the same time.

#### 3.2.3. Approaches Based on Graphs

The principle of this type of algorithm is to construct a graph gathering all the pairs of homologous genes shared by the compared genomes. These approaches aim at solving many problems raised by the methods presented before, in particular the possibility of studying several large genomes and to detect non-overlapping syntenic blocks. The previous approaches have difficulties decoding more complex genomic architectures that have undergone phases of significant duplication followed by reploidization. The search for non-overlapping syntenic blocks is of great interest because it makes it possible to focus on rearrangements that happened after the duplication events. However, the search for non-overlapping syntenic blocks is not just about simply decomposing overlapping blocks. Different algorithms have been proposed to meet these needs. The first algorithms as *GRIMM-Synteny* [183] or *MAGIC* [184] were suitable for small sets of genomes, but were not able to handle genomes with large duplications and deletions, and were not able to find non-overlapping blocks. Later, *Enredo* [169] was written to solve this problem. One last problem with the algorithms from the two previous approaches is that as more and more genomes are integrated into comparative studies, the number of genes shared between genomes decreases. This has a strong impact on the algorithms with the risk of rejecting the blocks because they are statistically nonsignificant, as it happens with tools such as *GRIMM-Synteny*.

Typically, the algorithms used in the graph-based approaches follow different steps. First, input genomes are locally aligned and the resulting alignments are used to construct a graph. Then, the different sub-structures (depending on the initial graph structure) are searched for to find the different segmentations of the genomes. The type of graph structure has a major influence on the results. Indeed, some of them handle these problems with more or less success and can therefore not find similar results. Four graph structures are predominant to analyze syntenic blocks.

The first structure corresponds to an alignment graph. The graph contains a vertex referring to each character in the sequence and edges referring to aligned characters. It is then possible to obtain collinear or noncollinear alignments by solving the maximum weight trace problem. Duplicated regions are more easily visible in an alignment graph structure. Nevertheless, this structure does not allow the user to get inversion information.

The second structure corresponds to A-Bruijn graphs that can be found in *DRIMM-Synteny* [168]. The main idea behind this graph is to merge aligned vertices. Thus, A-Bruijn graphs have one vertex for each aligned sequence. The links represent only the sequence. The main problem with this approach is represented by the short cycles, which tend to make local alignments hide a local collinearity. As an alignment graph structure, it does not allow access to inversion information, meaning that it is not possible to differentiate between the tandem repeats and palindromes.

The third structure, known as the Enredo graphs, can be found in *Enredo* [169]. It aims at managing genomes partitioned into segments. The nucleotide alignments are then made. Thus, Enredo graphs have two vertices per set of aligned segments, a head vertex and a tail vertex. It is then possible to eliminate various substructures from the Enredo graph in order to obtain the final segmentation of the genome. An Enredo structure can help find non-overlapping blocks and is suitable to consider non-overlapping inversions.

To finish, the Cactus graphs [185,186] have also been proposed. They are structures with vertices for adjacencies and undirected edges for genome segments. This type of graph is Eulerian meaning that there is a path that crosses all the nodes only once. This graph is also subdivided into independent units where each edge is part at most of one cycle. The cactus structure is a unique sub structure that allows an easy detection of short cycles.

These different graph structures allow the study of some particular sub-structures to identify syntenic blocks. One of the most important corresponds to the collinear paths. These sub-structures are a set of blocks that appear in genomes consecutively, without breaks and with the same orientation thus representing syntenic blocks. A second sub-structure corresponds to the presence of microblocks within larger regions that tend to introduce breaks into syntenic blocks. A third sub-structure corresponds to the short cycles. They are the mark of rearrangement. Indeed, similarities between sequences make them appear and thus break the collinearity. An important number of short cycles is problematic because they can aggregate into complex networks and hide true collinear blocks. We will detail a little bit more on one algorithm implementing the graph based approach below.

##### DRIMM-Synteny

This algorithm is the update of *GRIMM-Synteny* and aims at solving various problems from this previous version. In particular, the suppression of blocks due to their statistical non-significance in the case of the study of several distant genomes. The syntenic blocks that do not overlap are identified, which allows, in a second step, to bypass the threading problem based on the use of an A-Bruijn graph structure. This type of graph is an Eulerian and undirected multigraph. Edges are weighted by the number of times a gene pair is consecutive in the analyzed genomes.

In *DRIMM-Synteny*, an A-Bruijn graph is constructed by collapsing together identically labeled vertices from all genomes. From this graph, syntenic blocks can be found. In fact, a perfectly repeated block corresponds to a path in the graph. Perfectly repeated regions that do not share genes with other regions in the set of genomes being studied will appear as unconnected paths. These are referred to as the maximum paths in the graph, satisfying the condition that all of their internal vertices have only two neighboring vertices. This algorithm solves some existent problems known for this type of approach using different subroutines. There may be small differences between the different syntenic genes, which leads to short cycles. *DRIMM-Synteny* is able to detect them by computing a shaft at maximum range. A heuristic then allows detecting the links that create them in order to remove them. In addition, the presence of syntenic microblocks separate the long unbranched paths into several subpaths, thus complicating the detection of the blocks. Finally, the short palindromic regions that can be found within syntenic blocks form thornes that have the same effect as the microblocks.

### 3.3. Detection of Tandemly Arrayed Genes (TAGs)

Specific methods have been developed to handle specifically tandem duplication detection. TAGs are gene family members that are tightly clustered on a chromosome [73]. The vast majority of the methods are home-made pipelines available from the authors and may require programming skills. A few tools, particularly those related to the identification of syntenic blocks, are able to help in the identification of TAGs because they are generally summarized in a single occurrence of the dataset to lower the statistical noise. In general, they are not dedicated methods but more trivial algorithms. However, they have the advantage of being simpler to use. Most of these algorithms rely on protein comparisons, making them dependent on genome annotation. However, there exists very few methods that can deal with genomic sequences to search for long DNA tandem repeats. The advantage of these latest methods is that they can detect pseudogenes that originated from duplication or short ORFs generally missed by automatic genome annotation. We will first describe TAG detection in the genome at protein level, then at DNA level.

#### 3.3.1. Detection at Protein Level

These methods begin with the identification of homologous gene pairs. This can be done using different algorithms, in most cases an all-by-all BLASTP comparison of the proteome against itself or between the proteomes of two species, followed by a filtering using a threshold to retain only homologous pairs. The difference between these approaches lies in the homology assessment and the degree of sophistication to filter out false positives.

The most straightforward, but trivial, way is used in the first step of WGD detection algorithms such as *MCScanX* or *i-ADHoRe* [157,158]. These algorithms take as input homologous gene pairs, the preferred format being the BLASTP output. Then, the program classifies homologous pairs according to their rank along the chromosome. If consecutive BLASTP matches have a common gene and its paired genes are separated by fewer than five genes, these matches (forming a TAG) are collapsed using a representative pair with the smallest BLASTP e-value. The advantage of this approach is its speed but the drawback is that it can miss divergent homologous genes. Moreover, even if few programming skills are required, a parsing step is still necessary to obtain the list of identified TAGs.

To alleviate some problems related to the input (an all-against-all BLASTP), it is possible to use gene families as input. They can be constructed by different algorithms summarized in Table 1. Then, a TAG is defined as a block of adjacent genes belonging to the same family and separated by spacers that are generally genes not belonging to the homologous family. Several definitions can be used for the allowed number of spacers, mostly 0 or 1 but also ranging from 0 to 10 spacers [73,127,129,187]. The construction of gene families allows incorporating more distantly related homologs than the previous approach. The definition of homologous genes can be improved by merging all non-overlapping HSP of one hit [73]. The most widely used clustering algorithms are the single linkage algorithm, and more and more Markov clustering (MCL) and its variants. It is an efficient approach but adjusting the inflation and expansion parameters of MCL is not easy. The inflation parameter controls the flux between groups of classification (i.e., the number of steps in the random walk along the similarity graph). The expansion parameter controls the strength of links by strengthening them inside the clusters and weakening them between clusters.

#### 3.3.2. Detection at DNA Level

The vast majority of Tandem Repeat detection methods at DNA level deal with the identification of short highly repeated sequences. They are used to mask sequences corresponding to TEs or/and segments of low complexity before genome annotation or to explore the amplification of short duplications associated with human diseases for example, or copy number variation (CNV) between genotypes. These types of DNA duplication are not the focus of this review and will not be treated in detail. Here, we give a list of some famous short DNA Tandem Repeat detection tools able to deal with large datasets: *DUSTMASKER* [188], *SEGMASKER* [189], *Tandem Repeat Finder (TRF)* [190], *TANTAN* [191] and more recently *ULTRA* [192], *TARDIS* [193], and *dot2dot* [194].

We will now focus on long tandem duplication detection because all studies on TAGs based on protein similarity are biased by the quality of the available genome annotation. They exclude RNA genes or degenerated copies [195]. However, duplicated pseudogenes are an important evolutionary residue of a genome past activity [196]. A genome-wide approach has been proposed to take into account pseudogenes in TAG detection [197]. It scans, using TBLASTN, each protein against its chromosomal regions (the surrounding DNA sequences is three times longer than the CDS) and filter hits according to a refined bit-score, called the BTF score, that takes into account all non-overlapping HSP of less than 20% on the same strand. Then, it looks at CDSs in the ascending order of their chromosomal positions to extract TAGs. This mixed approach (at DNA and protein levels) is implemented in Python 2.4. The scripts are available from the authors and need a step of manual curation to eliminate false positive TAGs, due to the presence of minisatellites.

This previous approach is based on proteins and therefore depends on genome annotation. It has mainly been used on compact genomes [195]. *ReD Tandem* is an alternative method that circumvents this limitation [195]. Indeed, the main problem of detecting TAGs at genomic level is that large duplications despite being close, are far from being contiguous. The authors thus proposed to define tandemly duplicated segments as paralogous segments of size *l* with adjacent copies separated by a maximum distance *T* (in *A. thaliana*, the parameter values are *l* = 500 bp, *T* = 150 kb). The algorithm begins with anchors (paralogous segments of size *l*) and chains then using *DAGchainer* or *OSfinder* [175] into longer duplicated regions (called tandem units). Such alignable units are anchors of length *l* and separated by less than *L* bases (*L* = 40 kb for *A. thaliana*). Then, the tandem units are assembled into TAGs (i.e., tandem units separated by less than *T* bases, with *T* = 150 kb for *A. thaliana*). The C++ scripts are available but need some computational skills to be installed. Nevertheless, this elegant approach has allowed the authors to identify in *A. thaliana* several types of TAGs previously undetectable for genome-wide approaches. In decreasing order of importance, these new TAGs correspond to trans-elements genes, pseudogenes, pre-tRNAs, other RNAs, miRNAs, snoRNAs, and unknown genes [195].

### 3.4. Databases Storing Syntenic Block or Homology Information

#### 3.4.1. Syntenic Information

These databases have the advantage to not require computations and therefore no programming skills for the user. Some of them also offer visualizations and search tools. The main disadvantage is that they do not contain information from all organisms. Each of these databases provide particular features but some elements are common. In some cases, it is possible to access all the syntenic blocks between two organisms. The list of organisms is more or less extended depending on the database. Some of them propose to visualize these blocks using various representations such as circular visualizations, chromosome painting, or dot-plots. Some databases allow manually importing genomes to identify blocks of synteny. In this case, different tools may be implemented for the identification and are more or less easy to configure. For example, Ensembl [198] stores different information including syntenic blocks generated by Pecan [169] as a multi-alignment algorithm and *Enredo* to detect syntenic blocks. Synteny portal [199] and Genomicus [200] provide also syntenic blocks generated by *inferCars* [201] for different species but also multiple visualizations. Finally, other databases exist including ECRbase [202] with syntenic blocks generated from the DNA level. OrthoClusterDb [203] is a good example of what can be found inside these databases. Two main possibilities are available. First, it allows online access to the *OrthoCluster* tool [177] and to carry out identification of syntenic blocks on a remote server using a graphical interface facilitating the configuration and the retrieval of the results. Another possibility is to access different pre-computed syntenic blocks by *OrthoCluster* for different species. Pre-computed species belong to different groups (*Mammals*, *Pseudomonaceae*, *Drosophila*, *Plasmodium,* and *Caenorhabditis*) with 54 species available. The syntenic blocks can be visualized on a figure called genome painting which allows visualizing the chromosomes of the compared species with a system of colored segments highlighting the syntenic blocks. It is also possible to retrieve raw output files or to access to syntenic blocks using an online genome browser.

#### 3.4.2. Homology Relationships Databases

Dataset of duplicated genes without specification of the underlying mechanism of duplication can be retrieved from public databases. These databases can be associated or not with a specific methodology with available tools for a local use. The INPARANOID 8 database for example, provides the *InParanoid* tool and proposes orthology analysis between 273 proteomes, mostly eukaryotic. The dataset of orthologous and paralogous relationships between genes can be downloaded by pairs of species [204]. In HOGENOM, gene families are built from complete genomes from all three domains of life [147]. Its clustering pipeline is based on the *SiLiX* clustering method [205]. Even if this database is regularly updated, users can only retrieve families one by one according to keywords. The total amount of paralogous genes in a species is only available upon request directly to the authors.

Ensembl Compara is a specific section of the Ensembl database providing cross-species resources and analyses, at both the sequence and the gene levels. The main Ensembl database is dedicated to chordate genomes and displays now counterparts for several groups of organisms (Ensembl Genomes, Ensembl Bacteria, Ensembl Protists, Ensembl Fungi, Ensembl Plants, and Ensembl Metazoa). All these databases are associated with the Ensembl Compara system. This system provides access to protein gene families via a Perl API [198]. These families are built using all proteins from Ensembl through a classical process using BLASTP for similarity searches and a MCL clustering with scores as weight for edges in the initial graph. A final step aligns all sequences from a family using *MAFFT* [206]. It is to note that the Ensembl Compara protein families correspond to the most similar proteins compared to its gene tree section, where paralogous relationships are also available but in a tree format. Many other repositories are available but our goal is not to be exhaustive. Among the most generalist, we can cite PhylomeDB [148], OMA [207], OrthoDB [139], *OrthoInspector* [141], eggNOG [138], or the database Homologene from the NCBI portal.

For plant comparative genomic, we can cite the databases PLAZA [208], GreenPhyl [209], and Phytozome [210]. PLAZA 4.0 contains gene family data, phylogenetic trees, and gene colinearity information. It comprises two instances, one for monocots (Monocots PLAZA 4.5) that includes data from 39 species and one for dicots (Dicots PLAZA 4.0) that includes data from 55 species. The latest PLAZA instance offers one or more REST-full APIs, depending on the Platform software version. GreenPhyl 4 contains gene families and phylogenetic trees from 37 species. It has not been updated since 2015 but contains a section of manually annotated families comprising 2956 clusters. Other interesting sections are transcription factors and families specific to species or phylum (family of homologous genes found only in one species or excluding/including one phylum). Finally, the plant database Phytozome13 (last update in May 2019) contains 184 assembled and annotated genomes. Inparanoid pairwise orthology and paralogy groups have been calculated across all Phytozome proteomes and families of related genes representing the modern descendants of putative ancestral genes have been constructed at key phylogenetic nodes. The dataset can easily be downloaded or mined via a dedicated tool named *PhytoMine*.

## 4. Conclusions

To conclude, when considering duplicated genes inside a given species, it appears clear that they represent very different entities when taking into account their mechanism of formation, their fate, and their age. This is particularly important when it comes to their identification and analysis. It is indeed tempting to only detect all genes that are in several copies without taking into account the evolutionary complexity behind them. This is why it is also important to be aware of the different methodological approaches that can be used because this choice will greatly depend on the investigated biological question.

## Figures and Tables

**Table 1:** Estimation of the amount of duplicated genes in different species.

Species	No. of Considered Genes	No. of Estimated Duplicated Genes	% Estimated Duplicated Genes	Methodology	Duplicated Gene Types	References
*Arabidopsis thaliana*	25,557	11,937	46.7	All-against-all nucleotide sequence similarity searches using *BLASTN* among the transcribed sequences. Sequences aligned over >300 bp and showing at least 40% identity were defined as pairs of paralogs.	Not specified, all paralogous pairs were searched	[125]
	27,558	12,761	46.3 *	All-against-all protein sequence similarity search using *BLASTP* (e-value cutoff of e-10). Sequences alignable over a length of 150 amino acids with an identity of 30% were defined as paralogs. Gene families were built through single-linkage clustering.	Not specified, genes families were obtained	[69]
	25,972	10,483–17,406	40.4–67	All-against-all protein sequence similarity search using *BLASTP* (e-value cutoff of 1.0). For each pair of genes, blast-hits were merged to compute the total length and the global similarity of the aligned regions. Two datasets were constructed with respectively 30 and 50% sequence identity over respectively 70 and 90% protein length. Gene families were built through single-linkage clustering.	Not specified, genes families were all obtained (gene families)	[73]
	22,810	21,622	94.8 *	All-against-all protein sequence similarity search using *BLASTP* (top five non-self protein matches with e-value of 10e-10 were considered). Genes without hits that met a threshold of e-value 10e-10 were deemed singletons. Pairs of WGD duplicates were downloaded from published lists. Single gene duplications were derived by excluding pairs of WGD duplicates from the population of gene duplications. Tandem duplications were defined as being adjacent to each other on the same chromosome. Proximal duplications were defined as non-tandem genes on the same chromosome with no more than 20 annotated genes between each other. Single gene transposed-duplications were searched for from the remaining single gene duplications using syntenic blocks within and between 10 species to determine the ancestral locus. If the parental copy had more than two exons and the transposed copy was intronless, the pair of duplicates was classified as coming from a retrotransposition. Other cases of single gene-transposed duplications were classified as DNA based transpositions. Dispersed duplications corresponded to the remaining duplications not classified as WGD, tandem, proximal, or transposed duplications.	WGD, tandem, proximal, DNA based transposed, retrotransposed, and dispersed duplications	[48]
*Homo sapiens (human*)	33,869–>19,727	12,981	65.8	All-against-all protein sequence similarity search using *BLASTP* with the BLOSUM62 matrix and the SEG filter [126], *TribeMCL* with the default parameters. Tandem duplications were then searched for among families.	Gene families (tandem duplications searched among families)	[127]
	13,298	11,386	85–97	All-against-all protein sequence similarity search using *BLASTP* with cutoff expectation <2 and <10-e3.	Not specified, distant duplicates	[128]
	31,126	14,473	46.5 *	Ensembl family database and genes >300 nt. Tandem duplications were then searched for among families.	Gene families (tandem duplications searched for among families)	[129]
	20,415	15,569	76.3	Pooling of different datasets from [130] and all-against-all protein sequence similarity search using *BLASTP*.	WGD and SSD	[131]
	22,447	11,740	52.3 *	Ensembl version 77, >50% sequence identity, and high confidence for paralogy.	WGD and SSD	[74]
*Mus musculus (mouse)*	21,305	14,043	65.9	All-against-all protein sequence similarity search using *BLASTP* with the BLOSUM62 matrix and the SEG filter [126], *TribeMCL* with the default parameters. Tandem duplications were then searched for among families.	Gene families (tandem duplications searched for among families)	[127]
	27,736	16,091	58.01	Ensembl family database and genes >300 nt. Tandem duplications were then searched for among families.	Gene families (tandem duplications were searched for among families)	[129]
*Rattus norvegicus (rat)*	18,468	12,466	67.5	All-against-all protein sequence similarity search using *BLASTP* with the BLOSUM62 matrix and the SEG filter [126], *TribeMCL* with the default parameters. Tandem duplications were then searched for.	Gene families (tandem duplications searched for among families)	[127]
	27,194	16,446	60.48 *	Ensembl family database and genes >300 nt. Tandem duplications were then searched for among families.	Gene families (tandem duplications searched for among families)	[129]
*Oryza sativa* (rice)	18,562	9149	49.3	All-against-all nucleotide sequence similarity searches using *BLASTN* were done among the transcribed sequences. Sequences aligned over >300 bp and showing at least 40% identity were defined as pairs of paralogs.	Not specified, all paralogous pairs were searched	[125]
	42,534	8244–19,322	19.4–45.4	All-against-all protein sequence similarity search using *BLASTP* (e-value cutoff of 1.0). For each pair of genes, blast-hits were merged to compute the total length and the global similarity of the aligned regions. Two datasets were constructed with respectively 30 and 50% sequence identity over respectively 70 and 90% protein length. Gene families were built through single-linkage clustering.	Not specified, genes families were all obtained (gene families)	[73]
	27,910	21,461	76.9 *	All-against-all protein sequence similarity search using *BLASTP* (top five non-self protein matches with e-value of 10e-10 were considered). Genes without hits that met a threshold of e-value 10e-10 were deemed singletons. Pairs of WGD duplicates were downloaded from published lists. Single gene duplications were derived by excluding pairs of WGD duplicates from the population of gene duplications. Tandem duplications were defined as being adjacent to each other on the same chromosome. Proximal duplications were defined as non-tandem genes on the same chromosome with no more than 20 annotated genes between each other. Single gene transposed-duplications were searched for from the remaining single gene duplications using syntenic blocks within and between 10 species to determine the ancestral locus. If the parental copy had more than two exons and the transposed copy was intronless, the pair of duplicates was classified as coming from a retrotransposition. Other cases of single gene-transposed duplications were classified as DNA based transpositions. Dispersed duplications corresponded to the remaining duplications not classified as WGD, tandem, proximal, or transposed duplications.	WGD, tandem, proximal, DNA based transposed, retrotransposed, and dispersed duplications	[48]

* These values have been calculated according to the information provided in the corresponding reference article.

**Table 2:** Summary of the characteristics of different existing tools for identifying syntenic blocks.

Name	Input	Output Text	Output Plots	Main Algorithm	Specificities	Other Information		Documentation	Programming Language	Interface	References
						Gene Orientation	Genome Number				
*i-ADHoRe 3.0*	*BLASTP* output or gene families and list of genes in a gff like format	Tabulated text	Graphical visualization	Custom Greedy Graph	Typical implementation of the collinearity strategy	Yes	N	Complete	C++ Wrapper in Python	Command line interface	[158]
*MCScanX-Tranposed*	*BLASTP* output and a list of genes on chromosomes	Tabulated text	Graphical visualization	*DAGChainer* equivalent	Able to detect transposed gene duplications, detection of the type of duplicates	No	N	Incomplete and with errors	C++	Command line interface	[157]
*PhylDiag*	Species gene list and gene tree	Tabulated text	Graphical visualization	*DAGChainer* equivalent	Uses gene trees to define gene homologies. Takes into account gene orientations, and tandem duplication blocks	Yes	2	Complete	Python	Command line interface	[174]
*SynChro*	List of protein-coding genes and their associated amino-acid sequences	Text files containing homology relationships (RBH and non-RBH) and syntenic blocks description	Chromosomal painting representation, genome-wide dotplot	Computes Reciprocal Best-Hits (RBH) to reconstruct the backbones of the synteny blocks and complete with non-RBH syntenic homologs	Only one parameter: the synteny block stringency. Use *OPSCAN* instead of *BLAST* due to its optimization to detect RBH	only in visualizations	N	Complete	Python, bash	Command line interface	[160]
*Satsuma*	Nucleic sequences	Tabulated text	Multiple interactive plots	Cross-correlation, implemented as a fast Fourier transform	Based on a search strategy at a global level and cross-correlation at the local level	Yes	2	Short	C++, on linux	Command line interface	[155]
*DAGchainer*	Homologous genes and associated E-value	Tabulated text	Dot plot	Identification of chains of ordered gene pairs by searching paths in directed acyclic graph	Use of dynamic programming making it fast and highly reliable. Many softwares are based on this algorithm	No	2	Short	C++, Perl	Command line interface, Graphical user interface	[165]
*ColinearScan*	Any type of genetic markers (physical or genetic distance between markers, gene numbers)	Tabulated text with syntenic blocks and associated p-value	None	Dynamic programming algorithm based on the Smith-Waterman algorithm	Statistical inference, high computational efficiency, and flexibility of input data types	No	2	Not available	C++, Perl	Command line interface	[167]
*CYNTENATOR*	Sequences or alignments and an annotation file	Text file gathering alignments	None	Profile-profile alignment setting, which is an extension of the Waterman-Eggert algorithm	Implementing a phylogenetic scoring function	-	N	Complete	C++	Command line interface	[159]
*FISH*	List of the linear order and orientation of features on each contig andlist of the pairwise homologies between features	Text file results	Dot Plot	Dynamic programming algorithm based on the Smith-Waterman algorithm	Modeling of the probability of observing segmental homologies assumed by chance and taking this model into account to parameterize the algorithm and the statistical evaluation of its output	Yes	2	Not available	C++	Command line interface	[162]
*DRIMM-Synteny*	Set of anchors (e.g., local alignments or pairs of similar genes)	Text file where each genome is represented as a shuffled sequence of the syntenic blocks	Dot Plot	Construction of A-Bruijn graph	Graph-based algorithm allowing to identify non-overlapping syntenic blocks	No	N	Not available	C#	Command line interface	[168]
*DiagHunter*	*BLAST* output	Two text files containing gene names and/or coordinates	Dot Plot	Homology matrix based algorithm	Typical implementation of the colinearity strategy. Identifies large-scale syntenic blocks despite high levels of background noise	No	2	Short	Perl, and requires the BioPerl and GD.pm modules	Command line interface	[161]
*OSfinder*	Genomic locations of anchor or *BLASTP* results	Genomic locations of chains and orthologous segments	Dot Plot and a synteny map	Machine Learning and Markov Chains	Use Markov chain models and machine learning techniques. Automatically optimizes the parameters used in the Markov chain models. Scoring scheme based on stochastic models	Yes	N	Complete	C++	Command line interface	[175]
*SyMap*	Genome sequences in FASTA format and associated GFF files	Homologous genes, diagonals, and identified syntenic blocks.	Visualization available and interactive	*DAGChainer*	Interactive visualizations. Calculates synonymous and nonsynonymous mutation rates for syntenic gene pairs using *CodeML* of the *PAML* package	No	N	Complete	No requirements	Web user interface	[163]
*Cinteny*	Information about markers and the homologous groups.	Tabulated text	Three interactive visualizations Whole Genome Synteny, Chromosome Level Synteny, Synteny Around a Marker	Ternary search trees (TST)	On-the-fly computations allowing fast parameters adjustments	Yes	N	Complete	No requirements	Web user interface	[164]
*MultiSyn*	Protein sequences in FASTA format and genome annotation in BED	Output files from *MCScanX*	Multiple synteny plots	*MCScanX*	Efficient tool for non-programming skilled users. Precomputed data for 18 plant genomes	No	N	Not available	No requirement	Web user interface	[176]
*OrthoCluster*	Genome file and a file storing orthologous relationships among genes in all input genomes	Cluster file, with all the syntenic blocks detected, Stat file with information related to the size distribution of the syntenic blocks	One associated plot	Depth-first search method, can also use *Cinteny* or *SyMap*	Fast and easy to use. Can be applied using any types of markers as an input as long as their relationships can be established	Yes	N	Complete	C++	Web user interface, Command Line	[177]

N: Theoretically arbitrary number of studied genomes.
